# Unraveling Structure–Strain–Defect Relationships in Thermopower Modulation of Epitaxial Double Perovskite Oxide

**DOI:** 10.1021/acsomega.5c08622

**Published:** 2025-12-09

**Authors:** Arindom Chatterjee, Emigdio Chavez-Angel, Belén Ballesteros, Clivia M. Sotomayor Torres, José Santiso

**Affiliations:** † 16379 Catalan Institute of Nanoscience and Nanotechnology, CSIC and BIST, Campus UAB, Bellaterra, Barcelona 08193, Spain; ‡ ICREA—Institució Catalana de Recerca i Estudis Avançats, Barcelona 08010, Spain

## Abstract

Epitaxial strain engineering in oxide thin films offers a powerful strategy for tuning the electronic and thermoelectric properties by modulating the defect density, electronic band structure, and phonon scattering. In this study, we show that the gradual relaxation of in-plane epitaxial strain in layered double perovskite GdBaCo_2_O_5.5+δ_ thin films grown on SrTiO_3_ (001) substrates leads to a pronounced enhancement in thermoelectric power near the critical film thickness, where the epitaxial strain begins to relax, and a structural phase transition begins to occur. Our analysis suggests that this enhancement arises from an increase in carrier effective mass, likely caused by the reconstruction of electronic bands.

## Graphical abstract

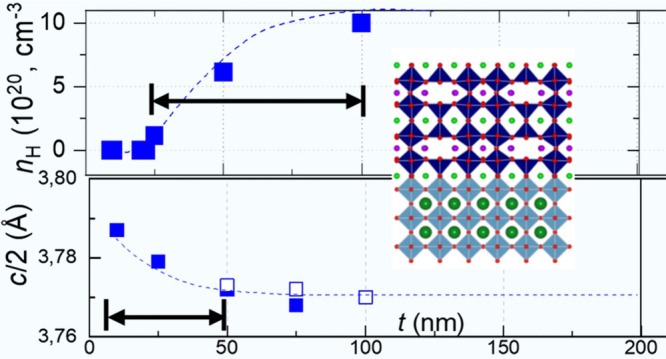

## Introduction

Harnessing waste heat through thermoelectric (TE) energy conversion has gained significant attention in recent years. Among various approaches, micro-TE devices have emerged as a promising platform for powering compact, next-generation technologies.^1,2^ Moreover, their compatibility with standard microfabrication techniques supports scalable application-specific designs. Notably, micro-TE devices based on materials with relatively low bulk thermoelectric figure of merit (zT), such as silicon, have demonstrated performance on par with state-of-the-art BiTe-based systems.^3–5^ These developments underscore the importance of advancing material-level strategies to further enhance device-level efficiency.

At the core of thermoelectric materials research lies the challenge of optimization and interrelation of charge carrier concentration (*n*), mobility (μ), and modulation of density of states (DOS) near the Fermi level (*E*_*F*_), as well as electronic band structure. These factors fundamentally govern the electronic conductivity (σ), the electronic part of the thermal conductivity (κ_*e*_), and the thermoelectric power (*S*) through the density of carriers, which together determine the overall thermoelectric performance.^6^ From a solid-state chemistry perspective, understanding and manipulating the structure-defect-property relationship are therefore essential to achieving controlled and enhanced transport behavior.

Among the various classes of efficient and promising thermoelectric materials, complex oxides stand out as strong candidates due to their pronounced structure–composition–defect–property relationship, which can be systematically manipulated in bulk and epitaxial thin films.^7,8^ In particular, epitaxial strain engineering, achieved by growing thin films on lattice-mismatched substrates, offers a powerful experimental tool to modulate the crystal symmetry and defect formation energetics in perovskite materials. These structural changes, in turn, have profound implications for the nature of charge carriers, DOS, and scattering mechanisms, all of which impact the thermoelectric properties.^9–16^

In this work, we focus on epitaxial thin films of double perovskite oxide GdBaCo_2_O_5.5+δ_, a class of materials known for their strong crystallographic anisotropy,^17^ cationic and oxygen-vacancy ordering,^17–20^ low chemical expansion^21^ and ability to exhibit both *p*- and *n*-type thermopower in both bulk crystals and epitaxial films.^22^ We report the pronounced enhancement of the thermoelectric power of epitaxial GBCO films by strain engineering. In particular, we investigate how the gradual relaxation of strain affects the structural, morphological, interfacial, electronic transport, and thermoelectric properties, uncovering a pronounced peak in performance near the critical thickness for strain relaxation. The insights from this study not only deepen our fundamental understanding of the structure–strain–defect relationship in complex oxides but also open new pathways for strain-tuned design of oxide thermoelectrics. This approach holds promise for scalable, high-efficiency energy harvesting technologies based on abundant and environmentally friendly materials.

## Results

Figure 1 shows the crystal structure of GBCO and the quality of our GBCO/STO films, which were fabricated by the pulsed-laser deposition (PLD) technique. GBCO features a layered perovskite structure, characterized by alternating stacks of GdO_*x*_ (in purple color) and BaO layers (in green color) along the crystallographic *c*-axis, sandwiched between CoO_2_ layers. The stacking sequence can be described as −[GdO_*x*_–CoO_2_–BaO–CoO_2_]–. This structure is known as a double perovskite, with Gd (0.50) and Ba (0.50) ions occupying the A-sites and Co ions occupying the B-sites in the ABO_3_ perovskite lattice.

**Figure 1:**
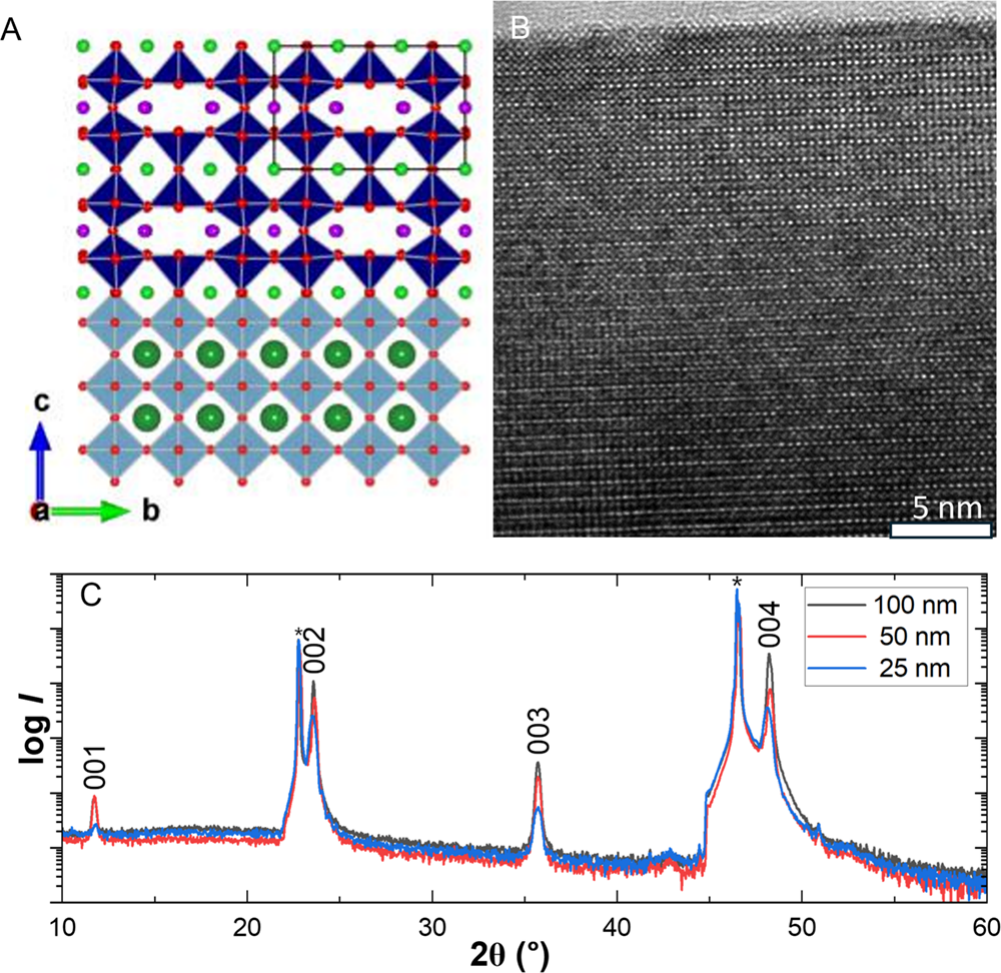
Crystal structure and epitaxy. (a) Pictorial representation of the epitaxial GdBaCo_2_O_5.5+δ_ (GBCO) films on SrTiO_3_ (100) (STO) substrates, (b) high-resolution TEM images, and (c) standard out-of-plane X-ray diffraction patterns.

The layered sequence of cation ordering along the *c*-axis is clearly visible in the high-resolution transmission electron microscope (HRTEM) image in Figure 1B. The cross-sectional TEM was taken on a 42 nm thick GBCO/STO film. The spacing between two consecutive bright layers is approximately 7.522 Å, which is twice the out-of-plane unit cell length of the primitive perovskite unit cell 2 × 3.761 Å, confirming the expected *c*-axis stacking. From our observations, we find no evidence of antisite disorder. The film domains display well-defined cation ordering, extending continuously from the substrate interface to the surface.

The standard out-of-plane X-ray diffraction (XRD) patterns, shown in Figure 1C, reveal the presence of 00L odd- and even-symmetric reflections. This clearly suggests that the GBCO/STO films are highly *c*-axis-oriented and maintain cation ordering throughout the film volume. Weak reflections observed around 2θ = 11°, 43°, and 51° in Figure 1C are not attributed to the film but arise from instrumental contributions, as discussed in detail in the Supporting Information Figure S1.

The methodology and the experimental setup for the in-plane thermoelectric power measurements are shown in the experimental section and in the Supporting Information (Figure S2, SI). Figure 2A shows the temperature and thickness dependence of the thermopower. In general, films of all measured thicknesses show thermal activation-like behavior between 250 and 350 K, i.e., thermopower decreases with increasing temperature. Specifically, the 100 nm film exhibits a thermopower of +60 μV/K at room temperature, which increases steadily with decreasing temperature, reaching a peak of +335 μV/K near 190 K before declining. The 250 nm film follows a very similar thermopower trend as a function of temperature, with nearly indistinguishable behavior. In contrast, the 50 nm film displays a much steeper increase, starting at +112 μV/K at room temperature and rising to approximately +440 μV/K around 200 K. Conversely, the 25 nm film shows a significantly more gradual increase in thermopower with decreasing temperature compared to the other three films. It is important to note that the temperature-dependent thermopower behavior of our GBCO/STO films closely resembles that of the *p*-type single crystals.^23^ However, the magnitude of the thermopower is very sensitive to the oxygen stoichiometry within the crystals.

**Figure 2:**
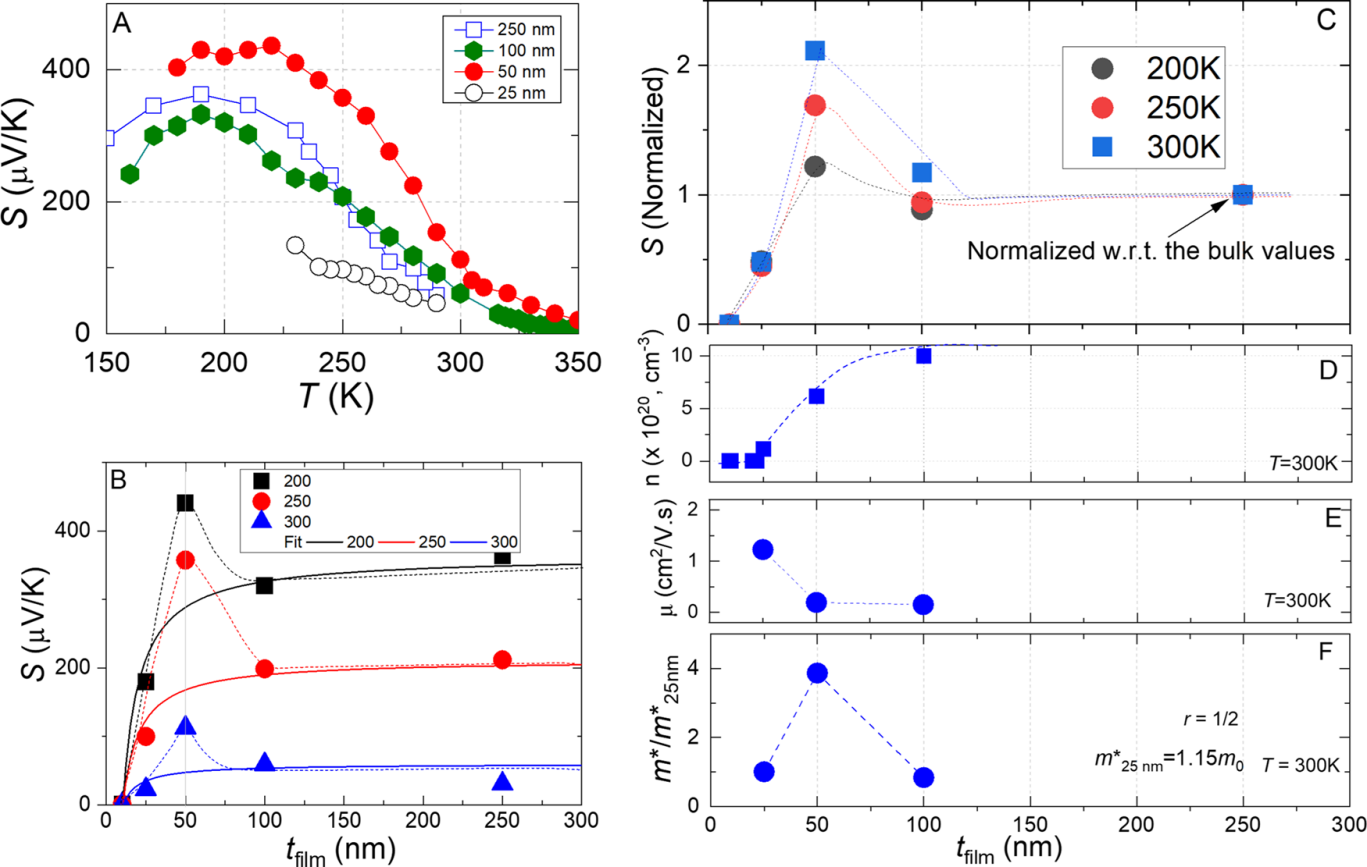
Thermoelectric properties of GBCO/STO films. (A) Temperature and thickness dependence on thermopower. (B) Thickness dependence on thermopower at fixed temperatures. The dotted lines are the observed trend, and the solid lines are fits. (C) Normalized thermopower as a function of film thickness. (D–F) Thickness dependence on the carrier concentration, carrier Hall mobility, and effective mass, respectively, at 300 K.

To better visualize the trend, we plotted thermopower as a function of film thickness at fixed temperatures (T = 200, 250, and 300 K; see Figure 2B,C). The data reveal a nonmonotonic dependence of thermopower on thickness. Notably, the 250 nm thick GBCO/STO film exhibits a thermopower of approximately +200 μV/K at 250 K, a value that remains nearly constant down to a thickness of 100 nm. However, as the thickness is reduced from 100 to 50 nm, the thermopower increases significantly, peaking at +358 μV/K. Further reduction to 25 nm leads to a sharp drop in thermopower, falling from +350 to +95 μV/K. For films thinner than 10 nm, the thermoelectric voltage becomes negligible, making accurate measurements difficult. This monotonic trend is consistent across all measured temperatures, where the films show thermal activation-like behavior. In addition, a thermopower peak of about +440 μV/K is observed at 200 K for a 50 nm thick film. It appears that the thermopower peak is most pronounced at a thickness of 50 nm (Figure 2C).

To assess whether the observed thermopower trend is linked to the charge carrier concentration, we performed Hall-effect measurements at 300 K. Figure 2D displays the estimated Hall carrier concentration as a function of the film thickness. A steady decline in carrier density is evident as the thickness decreases from 100 to 25 nm, spanning nearly an order of magnitude: from 9.8 × 10^–20^ cm^–3^ for the 100 nm film to 1.2 × 10^–20^ cm^–3^ for the 25 nm film. Films thinner than 20 nm exhibit insulating behavior, which aligns with the presence of a nonconductive interfacial dead layer, estimated to be approximately 17 (±2.3) nm thick (see Figure S3). These results indicate that the thermopower trend does not directly follow the carrier concentration.

## Discussion

It is important to note that previous studies on perovskite oxides have shown that the thickness of the electronic dead layer is independent of doping concentration.^24^ Thus, it is not affected by the intrinsic carrier concentration. Performing a comparable, systematic, doping-dependent investigation in GBCO is outside the scope of this work. Nevertheless, in our GBCO films, where the carrier density changes with thickness, we anticipate that the dead-layer thickness remains essentially constant and insensitive to variations in carrier density, as shown in Figure S3c, Supporting Information.

Thermopower is generally inversely proportional to carrier concentration in semiconductors or band gap materials. The simple parabolic band (SPB) model predicts a systematic increase in thermopower with decreasing carrier concentration.^25^ In GBCO/STO, thermoelectric power increases with decreasing thickness from 100 to 50 nm at all measured temperatures and followed by a sharp decline between 50 and 25 nm. Interestingly, this occurs despite a continuous decrease in carrier with decreasing film thickness. This thermopower-thickness-carrier correlation in GBCO/STO allows us to project the expected trend of thermopower with carrier, as shown by the data points in Figure S4, Supporting Information. As can be seen, the observed trend deviates significantly from the expected one. Furthermore, the SPB model anticipates a change of approximately Δ*S* = 196 μV/K for a change of 1 order of magnitude in carrier concentration.^26^ We observe Δ*n* = 4.1 × 10^–20^ cm^–3^ and Δ*S* = 160 μV/K between 50 and 100 nm, while Δ*n* = 2.9 × 10^–20^ cm^–3^ and Δ*S* = 265 μV/K between film thicknesses of 25 and 50 nm at 250 K. These deviations clearly indicate the involvement of a more complex electronic mechanism as a function of thickness.

A plausible mechanism underlying this modification is the classical size effect, which describes how finite dimensions influence thermoelectric power in polycrystalline films.^27–30^ This model predicts a relationship between the thermopower of film (*S*_*f*_) relative to its bulk (*S*_*b*_), which is given by eq 1:

$$S_{f}\left(t\right)=S_{b}\left(1-\frac{3l}{8t}\left(1-p\right)\frac{U}{1-U}\right)$$ 1

where *l* is the electron mean-free-path (e-MFP), *t* is the thickness, *p* is the secularity parameter, and *U* is the correlation parameter. In this model, the e-MFP governs the electronic resistance, resulting in reduced conductivity in thinner films due to enhanced surface scattering—qualitatively consistent with our observations (see Figure S5, Supporting Information). Although conductivity generally decreases with decreasing film thickness, the trend is less pronounced than that observed for thermopower. Nonetheless, it provides a basis for estimating an initial value for the electron mean-free-path.

Similarly, the thermopower is constrained by e-MFP and energy-dependent scattering mechanisms (*U*). This model predicts a steady decrease in thermopower below a critical thickness.^28–30^ The continuous lines in Figure 2B depict the expected trend of thermopower as a function of film thickness at a given *l* = 81 nm and *U* = 0.25, assuming diffusive behavior of the electrons (*p* = 0). Part of our measured trend remains similar to this approximation but differs significantly near the thermoelectric power peak around 50 nm thickness. The e-MFP and correlation parameter were estimated from the best fit of eq 1 to the experimental data. The fitting was, simultaneously, done by concatenating the normalized experimental data (*S*_*N*_) and the fitting equation through a Kronecker delta function (δ) as follows:

$$S_{N}=S_{1N}(t,\;T=200\;K)+S_{2N}(t,\;T=250\;K)+S_{3N}(t,\;T=300\;K)$$

$$S_{f}\left(t\right)=s_{1N}\left(1-\frac{3l}{8t}\frac{U}{1-U}\right)\delta_{1,i}+s_{2N}\left(1-\frac{3l}{8t}\frac{U}{1-U}\right)\delta_{2,i}+s_{3N}\left(1-\frac{3l}{8t}\frac{U}{1-U}\right)\delta_{3,i}$$ 2

where *s*_1*N*_ = 364 μV/K, *s*_2*N*_ = 212 μV/K, and *s*_3*N*_ = 60 μV/K represent the normalized Seebeck coefficient at each respective temperature. The above analysis suggests that the nonmonotonic behavior of thermopower with film thickness may be associated with intrinsic material properties such as crystal structure, orientation, interfaces, and surface properties.

Misoriented growth during thin film deposition can introduce additional anisotropic effects into the in-plane electronic and thermoelectric properties. However, in our case, the films with thicknesses ranging from 10 to 100 nm are highly *c*-axis oriented and epitaxial throughout their entire volume. This is supported by the observation of odd-symmetric reflections in standard XRD (Figure 1C) and the contrast variations between the Gd and Ba atomic layers in HRTEM images (Figure 1B). Therefore, we do not expect crystallographic anisotropy to play a significant role.

To understand the underlying mechanism, we investigated potential changes in crystal structure, cell parameters, in-plane epitaxial strains, and surface and additional interface properties as a function of film thickness. Using a 2x Ge (220) monochromator, we conducted high-resolution 2θ–ω measurements within the 46–49° in 2θ range, covering the high-intensity (002) substrate and (004) film reflections (see Figure 3e). We observed significant changes in the film reflections, both in the 2θ position and peak intensity as a function of film thickness. The presence of Laue fringes on both sides of the (004) film reflections indicates a high-quality and uniform film lattice. As the film thickness decreases, we observed (i) a systematic left shift of the (004) film reflections in the 2θ angle, indicating an increase in the *c*/2-parameter; (ii) Broadening of the peak full-width-half-maxima (fwhm), decreasing out-of-plane domain size; and (iii) a decrease in the relative intensities of the (004) film reflections, proportional to the film volume.

**Figure 3:**
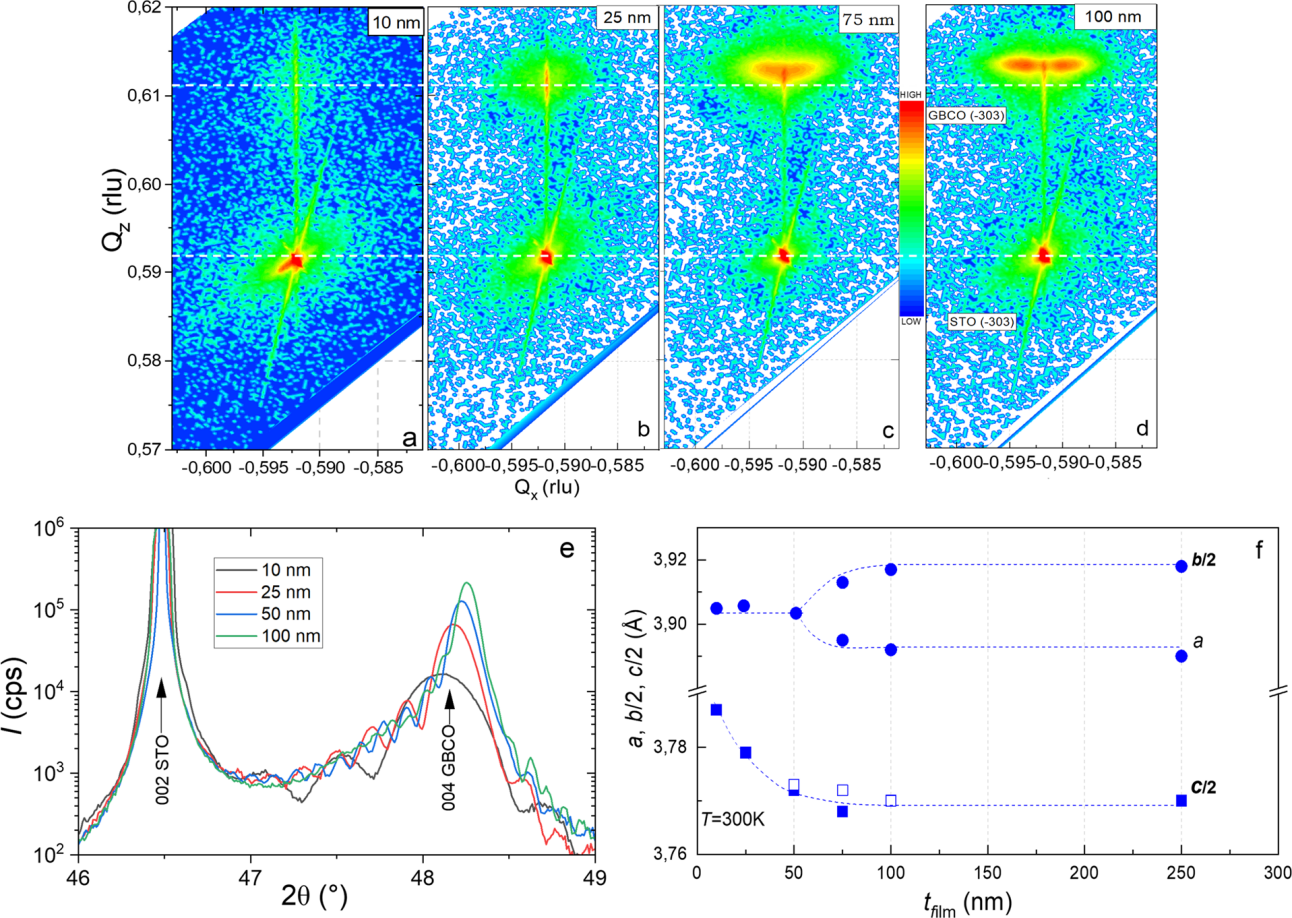
Strain relaxation and structural phase transition. (a–d) X-ray reciprocal space maps around (−303) reflection of GBCO with variable thicknesses on STO. Thickness-dependent evolution of (e) 004 reflection and (f) the in-plane and out-of-plane lattice constants of GBCO at room temperature.

A complementary comparison between the crystallite size estimated using the Scherrer equation and the film thickness obtained from XRR provides useful insight into the crystalline quality. In the ideal case of a fully coherent single-crystal film, the out-of-plane domain size from the Scherrer analysis would be expected to closely match the total film thickness. The results (Figure S6, Supporting Information) show a broadly consistent trend, but with noticeable differences in magnitude. These discrepancies can be attributed to the inherent limitations of the Scherrer equation, which is sensitive to factors such as point defects, strain gradients, mosaic spread, and the presence of multiple domains. The deviation observed for the thicker (100 nm) film suggests reduced crystalline coherence, resulting in an apparent thickness from XRD that is smaller than the thickness extracted from XRR.

In order to understand the in-plane mismatch, strain, and lattice constants, we performed high-resolution X-ray reciprocal space maps (RSMs). As shown in Figure 3a–d, the in-plane *Q*_*x*_ coordinate of the (−303) reflection of GBCO remains fully coherent with the substrate reflections (−303) for thinner films (10–25 nm). However, in 75 nm thick films, partial relaxation of the in-plane unit cell is observed. The 100 nm film shows a complete split into two lattice constants, indicating an orthorhombic structure. The 50 nm thick film, representing an intermediate thickness, still maintained coherent growth with the substrate. Furthermore, the RSMs reveal a contraction along the *c*-axis, evident from the shift in *Q*_*y*_ (indicated by the dotted line) axis as the film thickness increases from 10 to 100 nm, which agrees well with the standard high-resolution out-of-plane measurements. Table S1 depicts the in-plane strain calculations of GBCO films on the STO (100) surface. Overall, the high-resolution XRD analysis reveals a gradual relaxation of in-plane tensile strain as the thickness grows from 10 to 100 nm, transitioning from an effective +0.18% to a fully relaxed state. The effective strain was calculated with respect to bulk values *a* = 3.862, *b*/2 = 3.934, and *c*/2 = 3.786Å.^22^ Concurrently, a structural phase transition from a tetragonal to an orthorhombic phase is observed at room temperature, along with a progressive decrease in the out-of-plane parameter between 10 and 50 nm. The variations in cell parameters as a function of thickness are summarized in Figure 3f. Notably, we identified 50 nm thickness as the critical film thickness for structural phase transition, which coincides with the observed critical thickness for strain relaxation and thermopower peaks. This direct correlation strongly suggests a structural origin for the enhancement of thermoelectric performance.

On the other hand, as the in-plane domain splits up with increasing thickness, the interface density between GBCO and STO increases. Additionally, we observed a continuous increase in surface roughness from 0.6 to 1.7 nm as the thickness increased from 10 to 100 nm. An increase in both interface density and surface roughness suggests an increase in the density of scattering sites, which are likely to participate in scattering events and reduce charge carrier mobility. These events are expected to dominate above the critical thickness. The trend is evident in Figure 2E, where the 100 nm film shows lower mobility (0.15 cm^2^/V·s) compared to the 25 nm film (1.2 cm^2^/V·s) at 300 K. Notably, the most pronounced mobility drop occurs between 25 and 50 nm thickness, which coincide with the jump in thermopower (see Figure 2C) even though carrier concentration increases (see Figure 2D).

To further understand this apparent coupling of mobility and thermopower, we considered the role of the effective mass of electrons (m*). Since the mobility is inversely proportional to the effective mass and proportional to the scattering time (τ), we aimed to identify the dominant factor by estimating the effective mass of electron, independently from the mathematical relations shown in eqs 3–5.^31^ These equations enable the calculation of the reduced Fermi energy from the measured thermopower at a fixed temperature, assuming a scattering parameter of *r* = 1/2 in our case. Once the reduced Fermi energy is obtained, it can be used in eq 3 to estimate the effective mass, provided the carrier concentration is known.

$$S=\frac{k_{B}}{e}\left[\frac{\left(r+2\right)F_{r+1}\left(\eta \right)}{\left(r+1\right)F_{r}\left(\eta \right)}-\eta \right]$$ 3

$$F_{r}(\eta )=\int_{0}^{\infty }\frac{x^{r}}{1+e^{x-\eta }}dx$$ 4

$$n=(\frac{2\pi m^{*}k_{B}T}{h^{2}}{)}^{3/2}\frac{2}{\sqrt{\pi }}F_{1/2}\left(\eta \right)$$ 5

where *k*_B_ is the Boltzmann constant, *e* is the elementary charge of the electron, η is the reduced Fermi energy, *F* is the *n*th order Fermi integral, *h* is the Planck constant, m* = *m*/*m*_0_ is the effective mass of electrons, and *m*_0_ = 9.109 × 10^–31^ kg, the rest mass of the electron.

We independently measured both thermopower and carrier concentrations for our films and estimated the effective mass to be approximately 1.15*m*_0_^*^ for the 25 nm film. This value increases nearly four times that of the 25 nm film for the 50 nm film before decreasing to 1.25*m*_0_^*^ for the 100 nm film, as shown in Figure 3F. It is therefore very likely that the enhanced thermopower around the critical thickness is due to the increase in the effective mass of electrons.

Several mechanisms can lead to an enhancement of the effective mass, including band flattening, multivalley band structures,^32^ valence band convergence,^33^ or the polaron formation.^34^ First-principles (DFT+U) studies^35,36^ on GdBaCo_2_O_5.5_ reveal that its electronic structure is strongly governed by the spin state of Co^3+^ ions. Square-pyramidal Co^3+^ tends to adopt an intermediate-spin (IS) state, while octahedral Co^3+^ can stabilize in either low-spin (LS) or IS configurations, depending on the temperature and lattice distortions. These competing spin states lead to a narrow-gap insulating ground state and are responsible for the observed metal–insulator transition. The calculations further show strong Ising-type anisotropy and highlight how oxygen-vacancy ordering couples lattice distortions with orbital occupation, thus reconstructing the electronic bands and transport properties. Experiments on single crystals^17^ revealed pronounced Ising-like spin anisotropy and competing AFM-FM orders, highlighting the central role of oxygen vacancy-controlled spin states in defining the electronic and magnetic properties of GdBaCo_2_O_5.5_.

In our GBCO films, we do not observe any sharp insulator-to-metal transitions but rather a strong temperature dependence on electronic resistances (see Figure S7, Supporting Information). However, strain-driven oxygen vacancy concentration, ordering along the *c*-axis, and phase transition are evident. We believe that strain and oxygen vacancy ordering coupling assist with the spin-state transitions, which reconstruct the electronic structure associated with the structural phase transition. However, we do not have any direct evidence of any particular mechanism that can explain the increase in carrier effective mass. We propose that either the valence band convergence^33^ during the electronic reconstruction process or the formation of polarons enhances the effective mass^34^ due to strong electron–phonon coupling, as reported for standard perovskites. Two schematic illustrations regarding the proposed mechanisms are presented in Figures S8 and S9, Supporting Information.

In transition-metal oxides, the epitaxial strain accommodation mechanism is explained via oxygen vacancy formation, with tensile strain particularly favoring its formation.^37^ In the case of GBCO, oxygen vacancy ordering within the GdO_*x*_-layer influences the *c*-axis behavior. The competition between [Gd–Co–O] and [Ba–Co–O] blocks determines whether the unit cell expands or contracts along the *c*-axis, with the overall expansion occurring as oxygen stoichiometry increases,^38^ contrary to typical cell expansion in disordered perovskite upon oxygen vacancy increases. Notably, the *c*/2-parameter gradually decreases as the film thickness increases, at least from 10 to 50 nm (Figure 3f). In the absence of a change in the in-plane cell parameter between 10 and 50 nm thickness, the elastic response of the *c*-axis should also be constant. Therefore, the observed *c*-axis contraction is more consistent with increased oxygen vacancies. This implies that the mechanism of strain-induced structural phase transition is linked to the oxygen vacancy ordering. Consequently, the strong correlation among strain, defect, and structural phase transition results in the reconstruction of the electronic band structure during the process of the structural phase transition.

On the other hand, oxygen vacancies act as electron donors. In a *p*-type conductor, it compensates for holes and thereby reduces the density of *p*-type carriers. In our GBCO films, the *c*-axis lattice parameter decreases as the thickness increases from 10 to 50 nm, which is consistent with a reduction in oxygen concentration (i.e., increased oxygen vacancy). If oxygen stoichiometry were the dominant factor, one would, therefore, expect the *p*-type carrier density to decrease mainly within this 10–50 nm thickness range. In contrast, the Hall carrier concentration increases with increasing film thickness. In particular, it strongly increases between 25 and 100 nm. Furthermore, for thicknesses below ∼17 nm, the GBCO layers are electronically dead, while the *c*-axis remains expanded relative to thicker films. The fact that the structural evolution (10–50 nm) and transport evolution (50–100 nm) occur in different thickness regimes (see Figure S10, Supporting Information) indicates that the changes in carrier density are not directly controlled by oxygen-vacancy concentration but instead arise from an independent process.

A plausible explanation is the formation of a surface depletion region that suppresses the mobile carrier density in ultrathin films. Such thickness-dependent depletion effects are well established in transition-metal-oxide systems—including La/Nb-doped SrTiO_3_,^24,39^ where band bending and/or surface states near the free surface deplete carriers, so that the measured volumetric carrier density increases with increasing thickness as the relative contribution of the depleted region diminishes. Our *p*-type GBCO/STO films exhibit a similar trend, suggesting that surface depletion is largely independent of the carrier type and provides a natural explanation for the observed thickness dependence of the carrier density.

Since these structural and electronic transitions all occur near a film thickness of ∼50 nm, this regime likely represents a strain-stabilized intermediate state with a complex electronic structure. A comprehensive theoretical treatment of these effects, however, lies beyond the scope of the present study. Overall, our results show that strain relaxation as a function of film thickness is governed by a complex interplay of factors, including defects, structural phase transitions, interfacial roughness, interface density, electron mean free path, and crystallographic anisotropy. By optimizing the balance among the structure, strain, and defects, we were able to enhance the thermoelectric performance.

### Outline

In conclusion, our study reveals that the interplay among strain relaxation, structural phase transition, and defect dynamics plays a crucial role in governing the electronic band structure and thermoelectric properties of epitaxial films. By systematically controlling film thickness, we demonstrated enhanced thermopower near a critical regime, primarily driven by an increase in effective mass. These findings highlight the importance of nanoscale strain engineering and structural control in optimizing thermoelectric properties, offering a new design principle for next-generation oxide-based energy materials.

## Experimental Section

### Substrate Treatment

Prior to thin film deposition, we cleaned STO substrates in an acetone-bath sonicator for 15 min, followed by isopropanol for another 15 min. We dried the substrates with a nitrogen gun and placed them in the deposition chamber.

### PLD Parameters

We conducted all of the depositions under identical conditions. The substrate was heated to 850 °C at a ramp rate of 10 °C min^–1^ under an oxygen pressure of 60 mTorr. The distance between the target and substrate was maintained at 55 mm. A laser fluence of approximately 1.50 J cm^–2^ with a repetition rate of 10 Hz was used. Samples were cooled postdeposition under the same gas pressure with a ramp rate of 10 °C min^–1^. No postannealing treatments were applied to these samples.

### Thin Film Characterizations

Structural characterizations of the as-grown films were performed using a standard Panalytical X’Pert Pro MRD diffractometer with a 2θ–ω configuration. Standard XRD patterns were collected with and without a monochromator. Film thicknesses were determined from the X-ray reflectivity (XRR) and high-resolution 2θ–ω scans. In-plane and out-of-plane lattice constants, as well as epitaxial strains, were characterized using X-ray RSMs. The surface morphology of the thin films was examined using scanning electron microscopy (SEM) and atomic force microscopy (AFM). High-resolution TEM and HAADF-STEM images were acquired on an FEI Tecnai G2 F20 HR­(S)­TEM operated at 200 kV.

### Transport and Thermopower Measurements

Electronic conductivity and Hall-effect measurements were performed in a van der Pauw configuration from 50 to 300 K using a home-built cryostat. For Hall-effect measurements, the magnetic field was swept from −2 to +2 T in steps of 0.5 T. To measure the in-plane thermopower of GBCO/STO films, two thermometers (*T*_H_ and *T*_C_) and a heater were fabricated using optical lithography, followed by metal deposition, as illustrated in Figure S2 in the Supporting Information. A 5 nm chromium (Cr) layer was first deposited to promote adhesion to the oxide surface, followed by a 50 nm platinum (Pt) layer. A series of constant currents ranging from mAs was applied to the Cr/Pt metal strip, generating heat via Joule heating. This heat propagated laterally across the GBCO/STO film, establishing stable in-plane temperature gradients.

Thermoelectric voltages were measured under open-circuit conditions using a Keithley nanovoltmeter with a switch toggling between the *T*_H_ and *T*_C_ thermometers. The temperature coefficient of resistance for both thermometers had been calibrated beforehand at each fixed base temperature. To prevent current leakage, the Cr/Pt line heater was electrically isolated from the rest of the film.

### XRD and Analysis

GdBaCo_2_O_5.5+δ_ film crystals exhibit an orthorhombic structure at room temperature. We grew a series of GBCO films with varying thicknesses (10–250 nm) on STO (001) substrates using the PLD technique. Standard low-resolution 2θ–ω XRD patterns, obtained using a parabolic mirror and Ni filter (Figure 1C) and a 2× Ge (220) monochromator (Figure 3e). Substrate and film reflections are easily identifiable in the diffractograms: High-intensity, narrow peaks marked by asterisks correspond to the (00*L*) family of Bragg planes of SrTiO_3_ substrates, while the lower intensity, broader peaks marked by *hkl* Miller indices correspond to the GBCO film lattice.

## Supplementary Material



## Notes

Figure S1: X-ray diffraction with and without monochromator; Figure S2: Device layout; Figure S3: Dead-layer thickness; Figure S4: Thermopower vs carrier denisty; Figure S5: Thickness dependence on electronic conductivity; Figure S6: Film thickness vs out-of-plane domain size; Figure S7: Temperature-dependent electronic resistivity; Figure S8: Schematic illustration of the concept of valence band convergence; Figure S9: Schematic illustration of polaron formation and band flattening; Figure S10: Oxygen vacancy and carrier density relationship; Table S1: In-plane strain calculation (PDF)

## §

MESA+ Institute for Nanotechnology, University of Twente, Enschede 7500 AE, The Netherlands

## ∥

International Iberian Nanotechnology Laboratory (INL), Av. Mestre José Veiga s/n, 4715–330 Braga, Portugal.
