# Supplementary material for: Unraveling Structure–Strain–Defect Relationships in Thermopower Modulation of Epitaxial Double Perovskite Oxide
Source: ACS Omega. 2025 Dec 9;10(50):61891–8. doi: 10.1021/acsomega.5c08622 (PMC12750379; doi:10.1021/acsomega.5c08622)
Supplement: Supplementary file 1 [file ao5c08622_si_001.pdf]

Supporting Information

Unraveling Structure–Strain–Defect Relationships in Thermopower Modulation of Epitaxial Double Perovskite Oxide

Arindom Chatterjee,[*,‡] Emigdio Chavez-Angel,[‡] Belén Ballesteros,[‡] Clivia M. Sotomayor Torres,[‡,¶] and Jose Santiso[*,‡]

[‡]Catalan Institute of Nanoscience and Nanotechnology, CSIC and BIST, Campus UAB, 08193 Bellaterra, Barcelona, Spain
[¶]ICREA–Institució Catalana de Recerca i Estudis Avançats, 08010 Barcelona, Spain.
Current address: International Iberian Nanotechnology Laboratory (INL), Av. Mestre José Veiga s/n, 4715–330 Braga, Portugal
E-mail: arichatterjee1990@gmail.com ; jose.santiso@icn2.cat

Content:

1. X-ray diffraction
2. Device layout
3. Electronic dead-layer
4. Thermopower vs career concentration
5. Thickness dependence on electronic conductivity
6. Film thickness vs out-of-plane domain size
7. Temperature dependence on electronic resistivity
8. Valence band convergence
9. Polaron formation and band flattening
10. Oxygen vacancy-carrier density relationship
11. In-plane strain calculations

X-ray diffraction

Several weak peaks are observed around 2θ = 11°, 43°, and 51° in Figure 1C (main text). These peaks cannot be attributed to any known binary phases, yet they consistently appear across all samples, including bare substrates. The sharp feature near 51° is most likely related to residual Kβ radiation. Although the standard XRD measurements employed a parabolic mirror and Ni filter to suppress the Kβ contribution, the filter does not fully eliminate this component, and a weak reflection may still remain. In contrast, the peaks near 11° and 43° appear with similar intensities and shapes across all measurements, suggesting that they originate from instrumental contributions, likely associated with the Cu anode source and enhanced by the strong substrate reflections, rather than from the film. When using a primary beam 2 × Ge(220) monochromator, these contributions disappear. A difference in the enlarged XRD patterns with and without a monochromator is demonstrated in Figure S1.

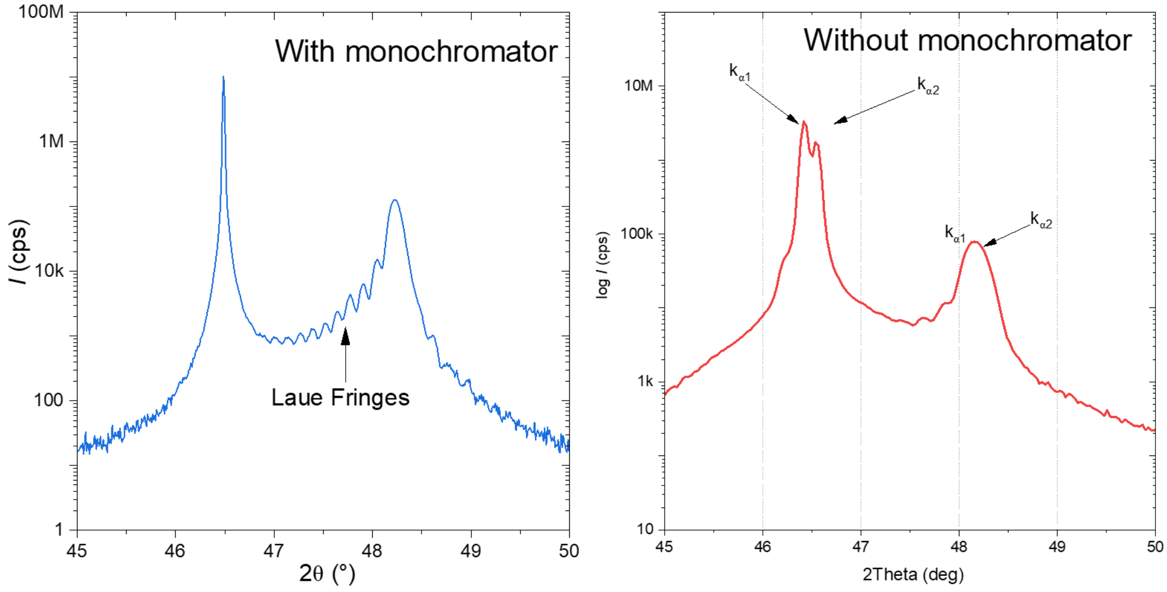

Figure S1. X-ray diffraction patterns of the GBCO/STO film with the monochromator (left) and without monochromator (right).

Device layout

To measure the in-plane thermopower of GBCO/STO films, we fabricated two thermometers (T_{Hot} and T_{Cold}) and a heater using optical lithography, followed by metal deposition, as illustrated in Figure S2. A schematic illustration of the device layout is shown in the left panel, and the actual mask design is shown in the right panel of Figure S2. In the actual device, two additional small pads were fabricated as backups for the Seebeck voltage measurements in case other electrodes were damaged during the measurement process. A 5 nm chromium (Cr) layer was first deposited to promote adhesion to the oxide surface, followed by a 50 nm platinum (Pt) layer. The temperature coefficients of resistance of the thermometers were measured at each fixed base temperature within ±10 K, since the difference in charge carrier density between the active layer and the metal electrodes varies significantly depending on the measurement temperature and film thickness. Thus, a single linear resistance-temperature (R–T) curve could not be generated. For calibration of the temperature gradients and thermoelectric voltages, the electrodes were designed over a relatively large sample area instead of a narrow Hall-bar geometry. Each four-point resistor/thermometer had a channel length of 1000 μm, and the separation for the thermopower measurement was 2500 μm. Thus, the effective area considered for thermoelectric characterization was 1000 × 2500 μm². A series of constant currents in the mA range was applied to the Cr/Pt metal strip (heater), which generated heat via Joule heating at one end of the substrate. This heat propagated laterally across the GBCO/STO film, establishing stable in-plane temperature gradients. Thermoelectric voltages were measured under open-circuit conditions using a Keithley nanovoltmeter, with a switch toggling between the T_H and T_C thermometers.

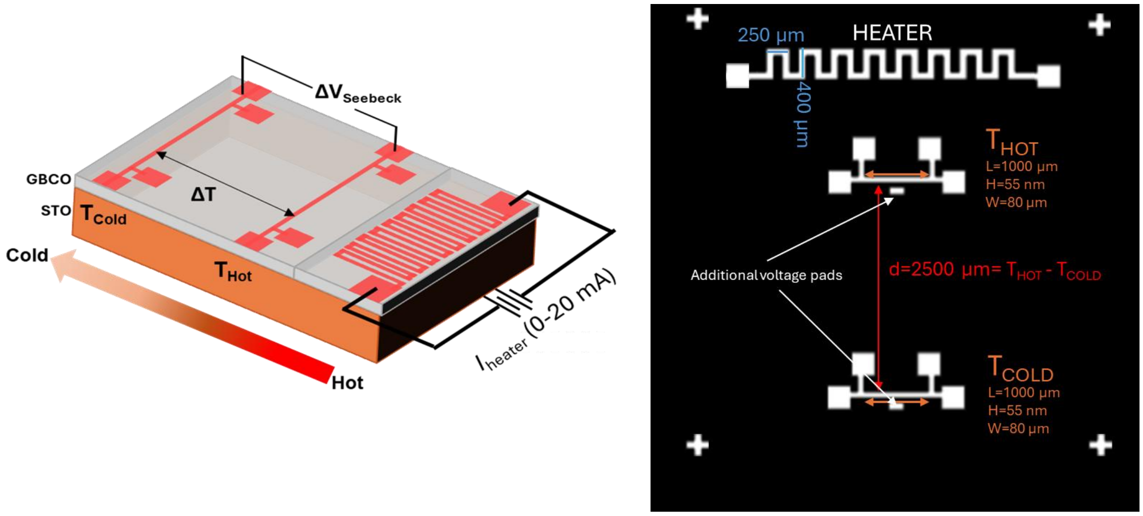

Figure S2. Schematic illustration of the device layout (left) and the dimensions of the electrodes (right).

Electronic dead-layer

A schematic illustration of the presence of an electronic dead-layer at the interface between GBCO and STO is shown in Figure S3a. This electronically inactive region is commonly observed in complex oxide heterostructures, where the first few unit cells at the interface do not contribute mobile carriers due to strain, interfacial disorder, or chemical mismatch. The thickness of the dead-layer can be estimated quantitatively from the sheet carrier density versus thickness plot, as shown in Figure S3b. In this representation, the linear increase of sheet carrier density with thickness allows one to extrapolate back to zero carrier density, thereby identifying the inactive thickness that corresponds to the dead-layer. Although this analysis clearly points to the existence of an interfacial dead-layer, it is noteworthy that the overall trend of the volume carrier density as a function of film thickness does not change dramatically. Instead, the evolution follows a consistent dependence across different thicknesses, suggesting that the effect of the dead-layer is confined to the interface region and its influence on the electronic response of the whole film volume remains uniform. Figure S3c further illustrates this comparison by plotting the thickness-dependent carrier density with and without considering the dead-layer, highlighting that while the absolute values shift, the functional trend is preserved.

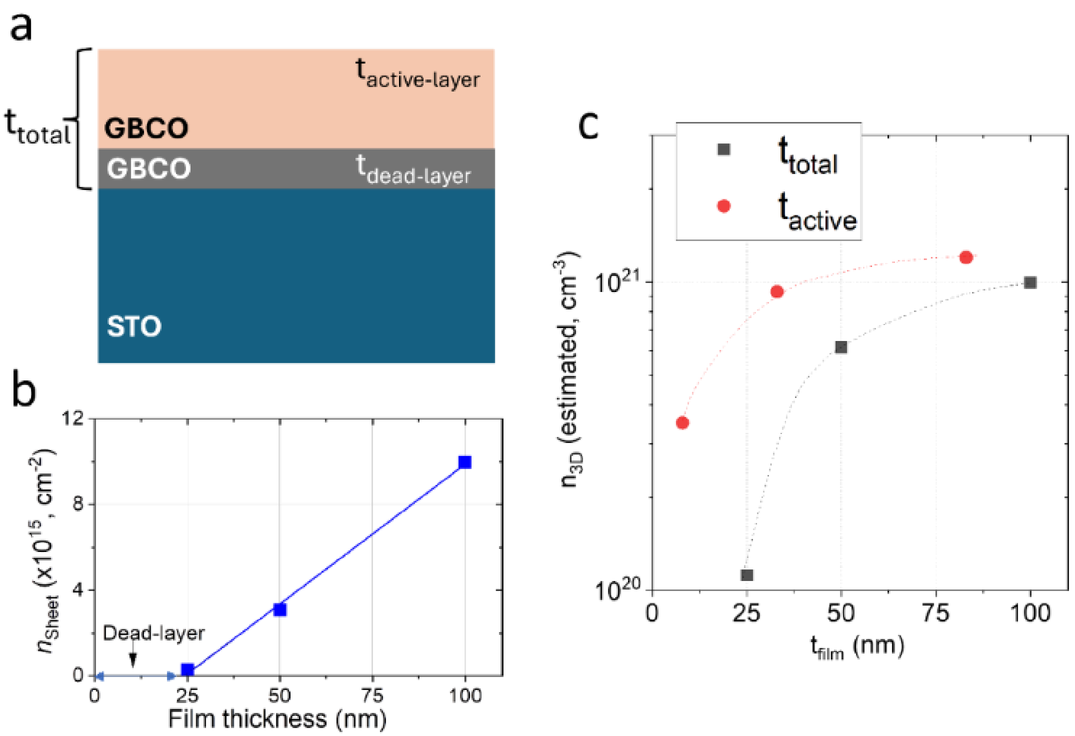

Figure S3. Dead-layer thickness. (a) schematic illustration of the electronic dead-layer between the GBCO film and the STO substrate. (b) estimation of the dead-layer thickness from thickness-dependent hall electron density measurements. (c) thickness-dependent carrier density with and without considering the dead-layer.

Thermopower vs career concentration

The single parabolic band (SPB) model anticipates a change in thermopower of approximately ΔS=196 μV/K for every one order of magnitude change in carrier concentration. This proportionality is derived from the fundamental relation S ∝ (kB/e) ln{*n*}, where k_B/e is the Boltzmann constant-to-electron charge ratio (≈ 86 μV/K), which sets the natural scale for thermopower changes in semiconductors. According to this relation, a logarithmic change in carrier density by a factor of ten should yield a predictable shift in Seebeck coefficient.

These deviations strongly suggest the presence of more complex mechanisms tied to film thickness, such as modifications of the electronic band structure, enhanced electron–phonon interactions, or contributions from defect states. Thus, while the SPB framework provides a useful baseline, the observed data highlight the critical role of epitaxial strain relaxation and defect ordering in governing the thickness-dependent thermoelectric response of the GBCO films.

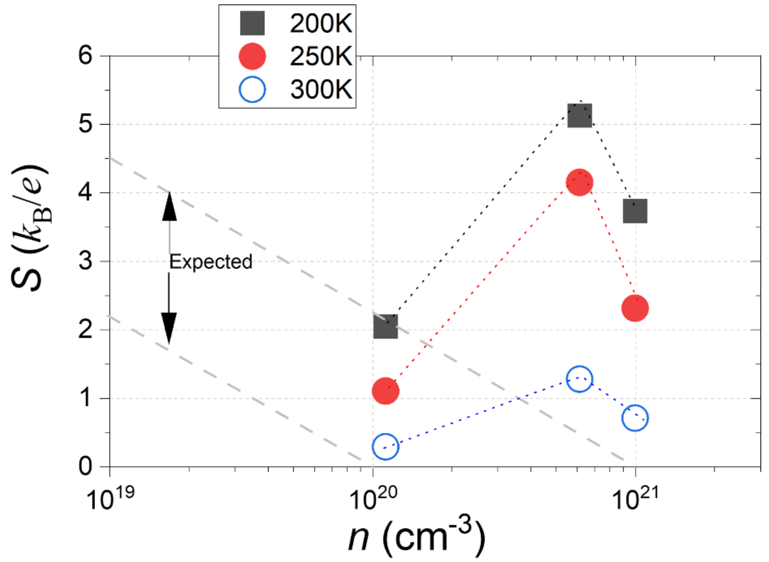

Figure S4. thermopower as a function of carrier concentration.

Thickness dependence on electronic conductivity

The thickness dependence of the electronic conductivity at 200, 250, and 300K for GBCO/STO films is shown in Figure S5. Although the conductivity values are presented over a wide range of film thicknesses, no systematic or monotonic trend can be clearly identified. This lack of a simple correlation reflects the fact that electronic conductivity is determined by two competing factors—carrier density and carrier mobility—both of which can vary independently with thickness. For instance, while the carrier density may increase with thickness due to reduced influence of the interfacial dead layer, the carrier mobility can be simultaneously affected by strain relaxation, defect scattering, or changes in microstructural quality. These competing effects make it difficult to isolate the dominant mechanism behind the observed variations in conductivity.

Even in the absence of a clear trend, these data are still valuable because they provide a framework for estimating the initial values of the mean free path of electrons in these epitaxial films.

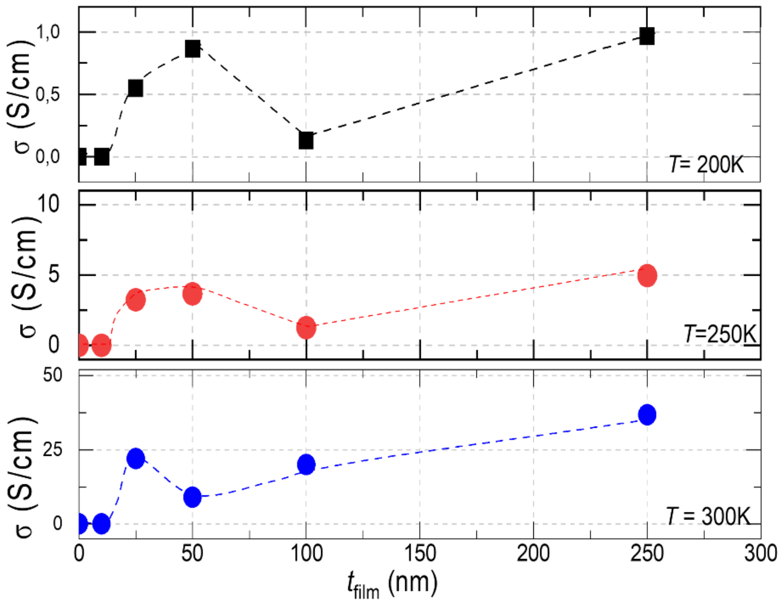

Figure S5. Thickness dependence on the electronic conductivity.

Film thickness vs out-of-plane domain size

The Scherrer equation reflects the size of coherently diffracting domains in the out-of-plane direction, and in the case of an ideal single crystal, this value should approach the total film thickness. Our comparison (Figure S6) shows a broadly similar trend but with clear differences in magnitude. These deviations are most likely due to the intrinsic limitations of the Scherrer analysis, which is sensitive to point defects, strain gradients, multiple domains, mosaic spread, and uncertainties in the assumed shape factor. Notably, the deviation becomes more pronounced in the thickest film (100 nm), indicating reduced crystal coherence; consequently, the apparent thickness derived from XRD pattern is smaller than that obtained from XRR. This interpretation is further supported by RSMs measurements, which confirm the presence of domains, strain relaxation, and structural phase transitions. Unlike the Scherrer analysis, which reflects only the size of coherently diffracting domains, XRR yields the total film thickness by probing the electron density profile and is therefore more accurate.

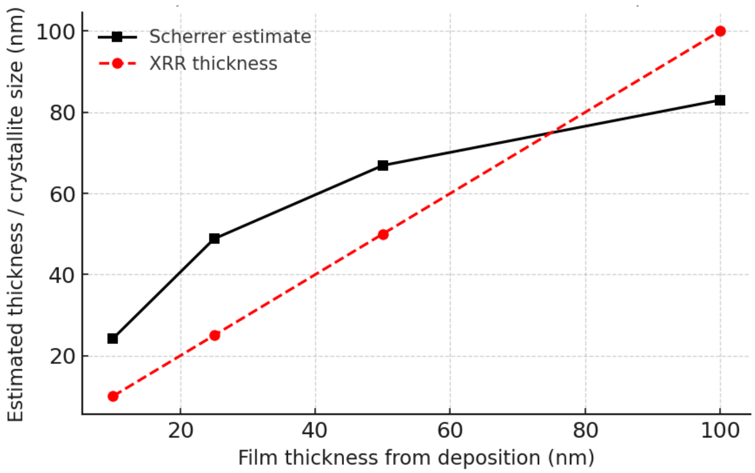

Figure S6. Comparison of the thickness determined from the X-ray reflectivity and domain size determination from the Scherrer equation.

Temperature dependence on electronic resistivity

In bulk GBCO crystals[1], oxygen vacancy ordering plays a crucial role in driving the orthorhombic–tetragonal structural transition, which strongly couples to the electronic states and gives rise to a sharp metal–insulator transition (MIT). The ordering of oxygen vacancies modulates Co valence and orbital occupancy, directly affecting charge transport. In contrast, epitaxial GBCO/STO films do not exhibit a well-defined MIT but instead show a pronounced temperature-dependent resistivity, suggesting that epitaxial strain, dimensionality, and partial suppression of vacancy ordering hinder a sharp transition. Temperature-dependent transport was measured in two regimes: 100–350 K in a cryostat under vacuum, and 350–1150 K in a tube furnace under controlled oxygen pressure, confirming the strong sensitivity of electronic properties to both temperature and oxygen environment.

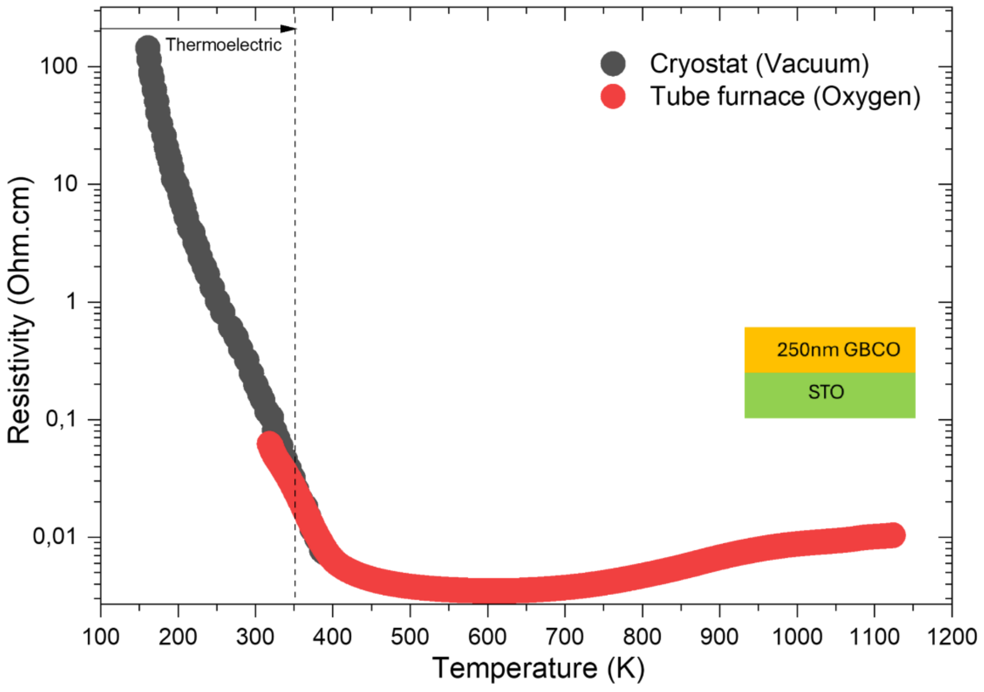

Figure S7. Electronic resistivity as a function of temperature, between 100 to 1150K.

Valence band convergence

Figure S8 schematically illustrates the concept of valence band convergence, where multiple valence band maxima shift closer in energy, effectively flattening the bands. This convergence enhances the density of states near the Fermi level, enabling higher Seebeck coefficients while maintaining good carrier mobility[2]. Such a mechanism increases the thermoelectric power factor by improving carrier transport efficiency without relying solely on higher carrier concentrations. Therefore, valence band convergence is considered a plausible pathway for optimizing electronic structure and enhancing thermoelectric performance.

In the case of GBCO, a similar principle can be linked to its structural phase transition between the orthorhombic and tetragonal states. Under epitaxial strain, the lattice distortion and subsequent strain relaxation drive this phase transition, which in turn modifies orbital overlap and band alignment. The strain-induced transition not only relaxes lattice mismatch but also facilitates electronic band restructuring, making band convergence a plausible mechanism that couples strongly to the structural evolution of GBCO films.

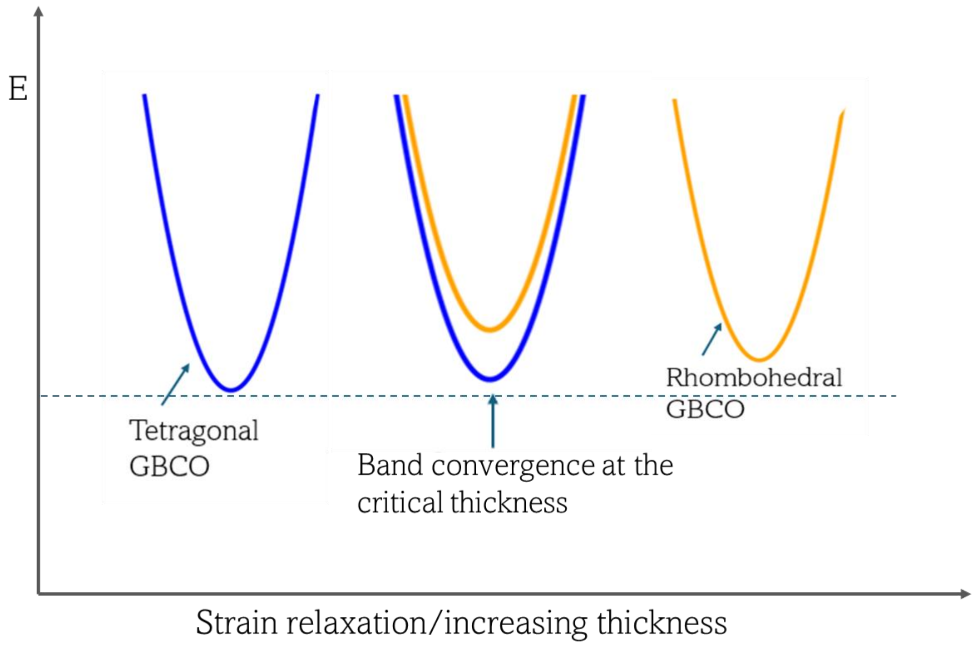

Figure S8. A schematic illustration of the concept of electronic band convergence.

Polaron formation and band flattening

Figure S9 schematically illustrates the concept of the polaron formation (left) and band flattening (right)[3]. Polaron formation arises from strong electron–lattice coupling, where local distortions increase effective mass and flatten the electronic band. This reduces mobility but alters electronic transport. In GBCO, such polaronic effects may be enhanced during the orthorhombic–tetragonal phase transition and strain relaxation, linking structural distortions with electronic band reshaping.

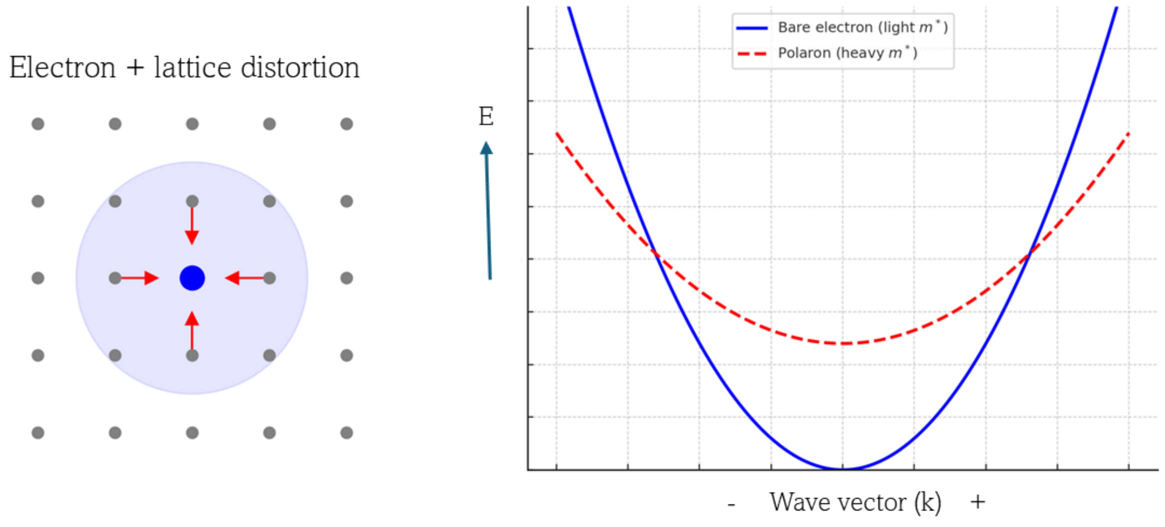

Figure S9. A schematic illustration of the formation of polarons and band flattening.

Oxygen vacancy-carrier density relationship

Figure S10 shows a comparison of the thickness ranges where significant changes occur in carrier concentration (top) and the *c*/2-parameter (bottom). The carrier concentration varies strongly between 25 and 100 nm, while films thinner than ~17 nm behave as electronic dead layers. In contrast, the c/2-parameter exhibits a pronounced and continuous increase between 10 and 50 nm, after which it saturates. Interestingly, although films below 17 nm are electronically inactive, the *c*/2-parameter still expands. Conversely, in the 50–100 nm range, the carrier concentration continues to change despite little to no variation in the *c*/2-parameter. This comparison clearly demonstrates that carrier density is not directly governed by oxygen vacancy concentration.

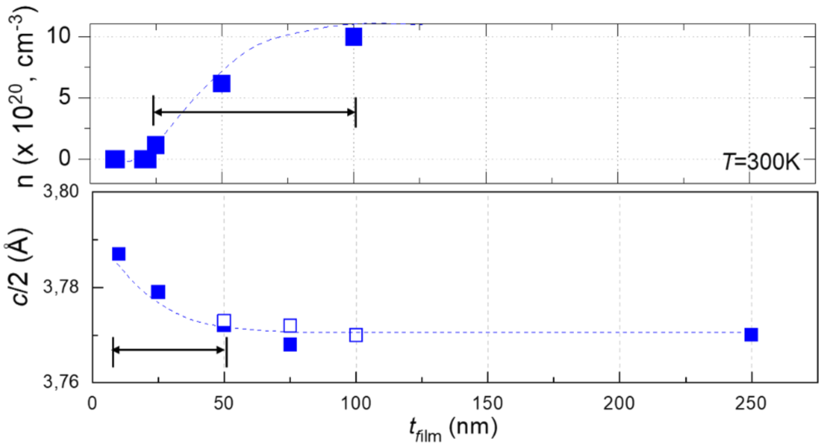

Figure S10. Comparison of thickness ranges showing significant changes in c-axis ordering and carrier concentration as a function of film thickness.

In-plane strain calculations

Table S1. Bulk reported lattice constants of orthorhombic GBCO are a=b/2=3.897 and c/2=3.786Å [4].

Thickness (nm)	Growth orientation/Matching plane	Effective (pseudo) in-plane of GBCO in Å	In-plane strain (%), related to bulk GBCO a=3.898Å
250	c-axis/a-b	=3.898	0 (fully relaxed)
100		$(\sqrt{(a \times \frac{b}{2})})$	0 (fully relaxed)
75			Partially relaxed
25			+0.18 (fully strained to STO)
10			+0.18 (fully strained to STO)

References

[1] Taskin, A. A.; Lavrov, A. N.; Ando, Y. Ising-Like Spin Anisotropy and Competing Antiferromagnetic–Ferromagnetic Orders in $GdBaCo_2O_{5.5}$ Single Crystals. *Phys. Rev. Lett.* **2003**, *90*, 227201.

[2] Pei, Y.; Wang, H.; Snyder, G. J. Convergence of Electronic Bands for High Performance Bulk Thermoelectrics. *Nature* **2011**, *473*, 66–69.

[3] van Mechelen, J. L. M.; van der Marel, D.; Grimaldi, C.; Kuzmenko, A. B.; Armitage, N. P.; Reyren, N.; Hagemann, H.; Mazin, I. I. Electron-Phonon Interaction and Charge Carrier Mass Enhancement in $SrTiO_3$. *Phys. Rev. Lett.* **2008**, *100*, 226403.

[4] Chatterjee, A.; Chavez-Angel, E.; Ballesteros, B.; Caicedo, J. M.; Padilla-Pantoja, J.; Leborán, V.; Sotomayor Torres, C. M.; Rivadulla, F.; Santiso, J. Large thermoelectric power variations in epitaxial thin films of layered perovskite $GdBaCo_2O_{5.5}$ with a different preferred orientation and strain. *J. Mater. Chem. A* **2020**, 8, 19975–19983.

Note: Each of these references is cited in the main text.
